# Vulnerability or resilience? Examining trust asymmetry from the perspective of risk sources under descriptive versus experiential decision

**DOI:** 10.3389/fpsyg.2023.1207453

**Published:** 2023-08-08

**Authors:** Jingyuan Zhu, Yingying Yao, Shan Jiang

**Affiliations:** Counseling and Education Center, Xiamen University, Xiamen, Fujian, China

**Keywords:** trust asymmetry, risk sources, natural risks, social risks, descriptive decisions, experiential decisions

## Abstract

**Introduction:**

The investigation of trust vulnerability is important to the understanding of the potential mechanisms of trust formation and erosion. However, more effective exploration of trust vulnerability has been hindered due to the lack of consideration of risk sources and types of information.

**Methods:**

This study extended the investigation of asymmetry to both social and natural risk under experiential and descriptive decisions. Using the trust game as the decision-making paradigm and money as the subject matter, the research employed experimental methods to examine how people perceive and make decisions after being positively and negatively affected by natural and social risks. A total of 286 college students were participated in our study. Study 1 (*n* = 138) and Study 2 (*n* = 148) explored asymmetry in experiential and descriptive decision separately.

**Results:**

The findings indicated that when considering experiential information, negative information had a greater effect in reducing trust compared to the enhancing effect of positive information (*t* = −1.95, *p* = 0.050). Moreover, the study revealed that negative information had a stronger negative impact in the context of social risks rather than natural risks (*t* = −3.26, *p* = 0.002), suggesting that trust is vulnerable both internally and externally. Conversely, when considering descriptive information, the effect of both positive and negative information on trust was symmetrical, and the impact of negative information was less significant compared to that of natural risks, indicating that trust has a certain level of resilience (*t* = 2.25, *p* = 0.028).

**Discussion:**

The study emphasizes the importance of refining risk sources and information characteristics in complex scenarios in order to improve understanding of trust enhancement and repair.

## 1. Introduction

The industrialization of society has brought significant wealth, but also introduced various risks, leading to a risk society characterized by multiple risks from various sources (Beck, 1992; Klinke and Renn, 2021). Technological risks include not only the associated effects of technological knowledge, but also the amplified risks arising from the misuse of modern technology for personal gain, particularly in the context of professionalized scientific activities where researchers receive funding and other economic benefits directly from corporations. Given the negative consequences of rapid technological advancements in the current risk society, which have increased public attention and debates on technological risks, discussing trust from the perspective of risk sources is critical.

The adage “trust goes on horseback and goes back on foot” indicates that trust is easier to be destroyed than to be built. While still relevant today, such discussions alone are insufficient in addressing complex trust issues in our current risk society. To understand the potential mechanisms of trust formation and erosion, especially the trust vulnerability, is beneficial to enhance risk management practices and reinforce public trust when dealing with intricate trust issues.

### 1.1. Trust asymmetry

Trust asymmetry refers to the asymmetry impact of negative events versus positive events on trust. The research on trust asymmetry can be approached from two perspectives: “information preference” and “mutual trust degree.” According to the information preference perspective, trust asymmetry occurs when negative information has a greater impact on shaping individuals’ trust levels than positive information (Beck, 1992; Klinke and Renn, 2021). The mutual trust degree perspective, on the other hand, sees trust asymmetry as a disparity in the level of trust held by different parties (De Jong and Dirks, 2012; Ran and Qi, 2019; Wang et al., 2020). The current study is looking from the information preference perspective of trust asymmetry.

Zucker’s (1986) research on trust and distrust laid the groundwork for the study of trust asymmetry based on information preference. Taylor (1991) and colleagues confirmed that people tend to place more emphasis on negative events, which reduces their trust (De Jong and Dirks, 2012). Slovic and her colleagues introduced the concept of “trust asymmetry” through their research in 1993 (Slovic, 1993). In this study, participants were asked to rate various descriptive traits based on behaviors that confirm or deny the traits. It turned out that positive traits require more behaviors to establish but easily lost, whereas negative traits are quickly established and difficult to dispel. Participants were also asked to rate the impact of news events concerning large nuclear power plants in their community on trust, with some information increasing trust while others decreasing trust. According to the findings, negative events had a much greater impact on trust than positive events, led to a conclusion that trust is asymmetric. Subsequent studies have confirmed the asymmetrical effect on trust (Siegrist and Cvetkovich, 2001; Cvetkovich et al., 2002; Poortinga and Pidgeon, 2004).

However, some studies on trust asymmetry draw to inconsistent conclusions. Researchers have examined various factors including the type of information presented, such as policy information versus event information (Cvetkovich and Winter, 2004), moral information versus intention information (Siegrist and Cvetkovich, 2001), and the type of disaster, such as high-risk, low-risk, or voluntary disasters (Slovic, 1993; Ran and Qi, 2019), to see how they influence trust asymmetry. Results showed that trust asymmetry is conditional, negative information does not necessarily have a greater impact on trust than positive trust-building experiences. Further research is required to fully understand the underlying vulnerabilities and resilience of trust.

### 1.2. Risk sources and trust asymmetry

Current research on trust asymmetry primarily concentrate on scientific and technological fields such as nuclear energy and pharmaceuticals. These studies aim to assess people’s trust in projects by evaluating the overall risks associated with these fields. However, the risks involved in these areas are highly intricate, and people have diverse concerns about them. To gain a better understanding of how trust is formed and eroded, it is necessary to break down the risks into more specific categories.

Some researchers introduced the concept of risk sources (Bohnet et al., 2008), classifying risks into two categories: natural risks, which are random and stem from the environment, and social risks, which trust is categorized as a type of, are caused by other people (Cvetkovich et al., 2002). Extensive research on trust asymmetry in social risks has been conducted while there is a notable lack of research examining the impact of information asymmetry in natural risks. The Prospect Theory introduced by Kahneman and Tversky (1979) highlighting the asymmetry in individual’s outcome preference, is considered as one of the main findings of asymmetries in decision making under natural risk. Besides, The House money effect in investment highlights the asymmetry in funding source preference when making risky decisions (Thaler and Johnson, 1990). Payzan-LeNestour and Doran (2022) found out that people are more likely to take risks when investing money that has been acquired through windfalls or effortless means, as opposed to money earned through hard work, which is usually invested more conservatively. It is necessary that the study of trust asymmetry be extended into other kinds of risks, such as natural risks.

Furthermore, only a few studies have looked into the dissimilarities in asymmetry between natural and social risks. Some study suggests that people respond differently to natural and social risks (Bohnet et al., 2008; Fetchenhauer et al., 2020). To improve our understanding of trust asymmetry, it is essential to conduct more comprehensive research from the perspective of risk sources, which could help bridge the gap between our understanding and the realities of trust asymmetry in the real world.

### 1.3. Experiential and descriptive information and trust asymmetry

The asymmetrical effects of natural and social risks may be influenced differently by descriptive and experiential information. Decision from descriptive information involves making decisions based on predetermined probabilities and outcomes (Kahneman and Tversky, 1979; Bohnet et al., 2008). This method of decision-making is commonly used in traditional decision research (Kahneman and Tversky, 1979; Thaler and Johnson, 1990). In contrast, decision from experiential information is a decision based on statistical probability, where subjects must obtain information about the probabilities and outcomes of each choice through their own experience before making a decision (Thaler and Johnson, 1990; Payzan-LeNestour and Doran, 2022). In recent years, experiential decision is getting more and more attention (Wulff et al., 2018). It was found that the differentiation effect between descriptive and experiential information is significant, a phenomenon known as the Description-Experience Gap (Hau et al., 2010; Garcia et al., 2021). When making choices based on experiential information, individuals are inclined to exhibit greater risk-taking behavior due to the sampling bias of experience and the influence of near-cause memory (Hertwig et al., 2004), when dealing with risks associated with safety, the time-delayed, abstract, and often statistical nature of the descriptive information does not elicit strong visceral reactions, while the recent personal experiences strongly impact the assessment of a risky option (Weber, 2006).

According to Chang et al. (2010), in terms of information processing and representation, the information in descriptive decision-making is predetermined and complete, leading individuals to adopt a more explicit and cognitive approach to decision-making. Conversely, the information in experiential decision-making is subjective and determined through experience, causing individuals to favor an implicit and emotional processing method. When individuals place trust in others and experience negative outcomes, the resulting sense of betrayal can lead to greater psychological distress than simple material losses (Bohnet et al., 2008; Humphrey and Mondorf, 2021). The impact of descriptive and experiential information on decision-making related to natural and social risks may differ. Therefore, it is important to explore the issue of trust asymmetry in both descriptive and experiential contexts.

### 1.4. Risk perception

Probability is a fundamental factor in decision-making when dealing with risks. People’s intuition regarding probability and randomness frequently deviates from statistical concepts (Incekara-Hafalir et al., 2021; Nobandegani et al., 2021). Subjective probability and risk perception refer to the individual’s personal assessment of external information related to social or natural hazards (Schmeidler, 1989; Falk and Konold, 1997; Lane, 2023), and may have a significant influence on the trust asymmetry. However, limited research has directly explored the role of risk perception in the asymmetry of trust under different circumstances. The present study seeks to address this gap in the literature.

### 1.5. The present study

The main aim of this study is to broaden the discourse on trust asymmetry by examining whether trust is asymmetric under both experiential and descriptive information, and whether it is more susceptible to negative information as a type of social risk compared to natural risks.

Specifically, the study aims to investigate trust asymmetry by analyzing how positive or negative information affects decision-making under both descriptive and experiential contexts. Decision-making based on descriptive information usually involves rational and explicit processing with less emotional influence, while experiential information typically involves implicit processes with greater emotional influence. Betrayal resulting from trust violations can have more severe emotional consequences than pure economic losses, suggesting that negative experiential information may lead to higher levels of trust asymmetry in social contexts compared to natural risks where the asymmetry caused by pure economic losses is less pronounced. Processing of descriptive information is less influenced by emotions, and positive and negative information have a more symmetrical effect on decision-making. Therefore, this study proposes the following hypotheses:

*Hypothesis 1*: Negative experiential information has a *greater* impact on reducing *social* risk adventure compared to positive experiential information increasing it.

*Hypothesis 2*: Negative experiential information has a *similar* impact on reducing *natural* risk adventure compared to positive experiential information increasing it.

*Hypothesis 3*: Negative descriptive information has *similar* impact on reducing *social* risk adventure compared to positive descriptive information increasing it.

*Hypothesis 4*: Negative descriptive information has a *similar* impact on reducing *natural* risk adventure compared to positive descriptive information increasing it.

We also intend to take a deeper look into how negative information influences decision-making n natural and social risk scenarios. Under experiential information conditions, the presence of psychological and emotional losses resulting from betrayal, which goes beyond monetary loss, may lead to a more significant impact on social risk than natural risk. Conversely, in descriptive decision-making, the confirmatory bias hypothesis proposes that individuals evaluate the credibility of external descriptive information based on their past experiences (Zucker, 1986; Slovic, 1993; Wang et al., 2020). People’s assessment of credibility in everyday situations serves as an anchor in the risk assessment of trust decisions that based on descriptive information. Based on this, the following hypothesis are proposed.

*Hypothesis 5*: Negative *experiential* information may have a *stronger* impact on social risk adventure compared to natural risk adventure.

*Hypothesis 6*: Negative *descriptive* information may have *weaker* impact on social risk adventure compared to natural risk adventure.

The study also aims to investigate the role of risk perception in the impact of positive or negative information on trust asymmetry, which leads to the last hypothesis of the study:

*Hypothesis 7*: Risk perception serves as a mediator in the phenomenon of trust asymmetry.

Two studies, Study 1 and Study 2, were conducted to investigate the impact of experiential and descriptive information on asymmetry, respectively.

## 2. Study 1: the impact of experiential information on asymmetry

This study aims to examine the impact of experiential information on both natural and social risky decision makings.

### 2.1. Method

#### 2.1.1. Participants

A group of 138 Chinese college students (71 males and 67 females) were recruited for this study. The mean age was 20.2 (*SD* = 1.45) years, ranging from 18 to 24 years. The exclusion criteria are (1) students major in psychology or economics, (2) had participated in similar decision-making tasks or study. All the participants in study 1 were randomly assigned to four groups based on experimental design, named Natural-Positive group (*n* = 31), Natural-Negative condition group (*n* = 34), Social-Positive group (*n* = 35) and Social-Negative group (*n* = 35).

#### 2.1.2. Design

A 2 (risk source: natural vs. social) × 2 (information valence: positive vs. negative) experimental design was employed in the study.

#### 2.1.3. Procedures and materials

The investigation utilized a Trust Game consisting of two rounds to serve as the decision-making task. The computer-based platform was used to implement the task and generate virtual partners for participants, without disclosing the virtual nature of the partners. Upon completion of the experiment, the participants were provided with small tokens of appreciation as a gesture of gratitude. The experimental procedure was composed of three distinct steps.

##### 2.1.3.1. Step 1

**Figure 1:**
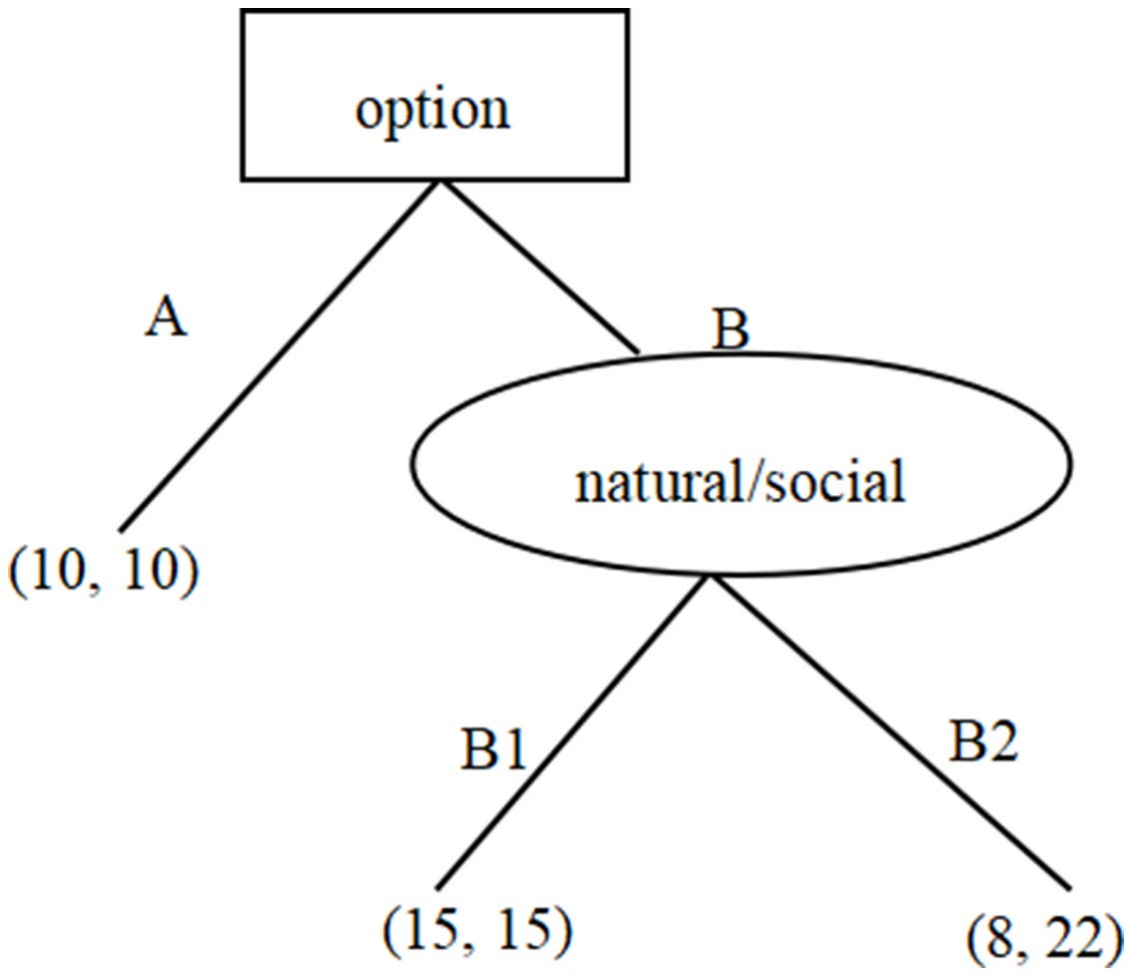
Task rules of trust game.

During the initial stage, participants engaged in the first round of the Trust Game, where they assessed the risks and made a decision. They were paired with an anonymous partner and presented with two options, labeled as option A and option B, as illustrated in Figure 1. Participants were required to select between the two options. The participants are informed of the following information: opting for option A resulted in both the participant and the partner earning ¥10 each (with the number before the comma indicating the participant’s earnings, and the number after the comma indicating the partner’s earnings), leading to the task concluding. Choosing option B resulted in a total income of ¥30, with two possible distribution outcomes: mutual ¥15 each (15, 15) as outcome B1, or ¥8 for the participant and ¥22 for the partner (8, 22) as outcome B2.

In the natural risk condition, the participants were told that the ultimate allocation outcome of B1 or B2 was established via a random drawing process. In contrast, in the social risk condition, they were told that the final allocation outcome of B1 or B2 was based on the choice of the partner. Self-reported risk perception and participants’ preference for option A or B would be collected. Half of the participants were required to make their decision before evaluating the risks, while the other half made their decision after completing the risk assessment process.

##### 2.1.3.2. Step 2

Feedback information was presented randomly to participants, irrespective of their selection of option A or B. Four distinct conditions were employed in the study.

The Natural-Positive condition: If the participant selected option B in Step 1, the feedback indicated “The final lottery result was (15, 15).” If the participant chose option A in Step 1, the feedback stated, “If you chose option B right now, the result of the draw would be (15, 15).”

The Natural-Negative condition: If the participant selected option B in Step 1, the feedback indicated “The final lottery result was (8, 22).” If the participant chose option A in Step 1, the feedback stated, “If you chose option B right now, the result of the draw would be (8, 22).”

The Social-Positive condition: If the participant selected option B in Step 1, the feedback indicated “The other party’s choice was (15, 15).” If the participant chose option A in Step 1, the feedback stated, “The opponent’s choice is (15, 15), and if you just chose option B, the result is this.”

The Social-Negative condition: If the participant chose option B in Step 1, the feedback stated “The other party’s choice was (8, 22).” If the participant opted for option A in Step 1, the feedback stated, “The opponent’s choice is (8, 22), and if you just chose option B, the result is this.”

##### 2.1.3.3. Step 3

Following the feedback presentation, participants proceeded to the second round of the trust game, where they evaluated risks and made decisions. Self-reported risk perception and participants’ preference for option A or B would be collected the second time. It’s noteworthy that participants were consistently exposed to the same type of risk throughout both rounds of the experiment, with each round involving either natural or social risk.

#### 2.1.4. Outcome variables

The study evaluated the impact of the interventions on two key outcomes: changes in self-reported risk perception and changes in adventure between the two rounds of the trust game. Adventure was measured on a 9-point scale, with a score of 1 indicating a complete preference for option A, 5 indicating neutrality, and 9 indicating a complete preference for option B. A higher score indicated a greater degree of adventure. Risk perception was measured by participants’ estimates of the probability of outcome B1 (15, 15) if they chose option B, with values ranging from 0 to 1. Higher values reflected lower risk perception. Under positive conditions, change in adventure and risk perception were determined by subtracting the first-round score from the second round score, while under negative conditions, change in adventure and risk perception were determined by subtracting the second round score from the first-round score.

### 2.2. Results

The order whether “evaluation before decision” or “decision before evaluation” was found to have no significant impact on the outcomes of risk perception and adventure. As such, this factor was not considered in further analysis. Results from the first round of the game showed no significant difference in risk perception and adventure between the positive (*M*_risk perception_ = 0.55 ± 0.27, *M*_adventure_ = 5.89 ± 2.69) and negative groups (*M*_risk perception_ = 0.58 ± 0.24, *M*_adventure_ = 5.87 ± 2.68), *p* > 0.05, indicating that the samples in both groups were comparable and unbiased in participant selection.

**Figure 2:**
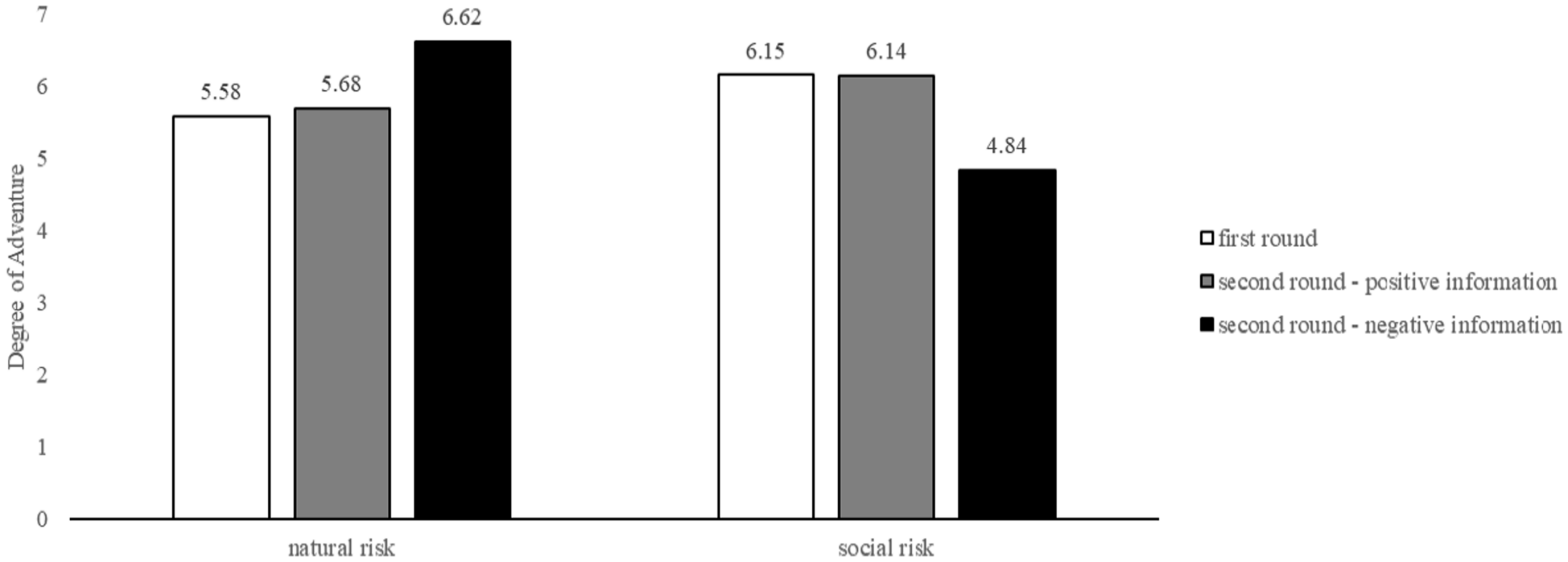
Degree of adventure under natural and social condition in first and second round.

The degree of adventure was observed under both natural and social risk conditions in the first and second rounds of the game. The results were depicted in Figure 2. There was no significant difference in adventure between natural and social risk in the first round of the game, *t* (136) = −1.24, *p* > 0.05. In the second round of the game, there was no significant difference in adventure between natural and social risks, *M_natural risk_* = 5.98 ± 2.62, *M_social risk_* = 5.63 ± 2.62, *t* (136) = 0.79, *p* > 0.05. Among those who received positive information, there was also no significant difference in adventure between natural and social risks, *M_natural risk_* = 5.68 ± 2.75, *M_social risk_* = 6.77 ± 2.07, *t* (64) = −1.84, *p* > 0.05. However, for those who received negative information, there was a significant difference in adventure between natural and social risks, *M_natural risk_* = 6.26 ± 2.51, *M_social risk_* = 4.58 ± 2.66, *t* (70) = 2.76, *p* = 0.007.

Table 1 provided a summary of the change in adventure and risk perception on natural and social risks after experiential information was given.

**Table 1:** Impact of experiential information on natural and social risks.

	Natural risk		Social risk	
	Change in adventure	Change in risk perception	Change in adventure	Change in risk perception
Positive information	0.26 ± 0.96	−0.01 ± 0.09	0.40 ± 1.22	0.03 ± 0.07
Negative information	−0.53 ± 2.25	0.02 ± 0.20	1.32 ± 2.53	0.11 ± 0.21

In natural risk, no significant change of positive or negative feedback on risk perception or adventure was found, with adventure *t* (63) =1.80, *p* > 0.05 and risk perception *t* = −0.51, *p* > 0.05. However, under social risk, the change of positive and negative information on risk perception and adventure was significant. Negative information led to a greater reduction in adventure compared to the increase in adventure resulting from positive information, with adventure *t* (71) = −1.95, *p* = 0.050, and risk perception *t* (71) = −2.31, *p* = 0.024. Both Hypothesis 1 and Hypothesis 2 have been supported by the results.

The study findings showed that negative information had a more significant impact on change of risk perception and adventure in the context of social risks compared to natural risks, with adventure *t* (70) = −3.26, *p* = 0.002, risk perception *t* (70) = −2.00, *p* = 0.049. The results provide support for Hypothesis 5.

An intermediary effect analysis procedure was applied to investigate the role of risk perception change in the impact of information valence on risk-taking under social risk, using the Bootstrap method proposed by Preacher and Hayes (2004) and Hayes and Scharkow (2013) with a sample size of 5,000 and a 95% confidence interval. The results revealed a significant intermediary effect of risk perception change, with a size of 0.942, and a confidence interval that did not contain 0 (LLCI = 0.254, ULCI = 1.733). This suggests that changes in risk perception play a crucial role in mediating the influence of information valence on trust. The confirmation of Hypothesis 7 was observed under the experiential context condition.

## 3. Study 2: the impact of descriptive information on asymmetry

Study 2 examined the influence of descriptive information on decision making under different risk sources.

### 3.1. Pre-experiment

The same trust game task as in Study 1 was used in Study 2, but with a descriptive decision-making paradigm. Prior to making their decisions, participants were informed of the probability of Option B’s outcome in percentage form. Under the positive information condition, participants were informed that there was an 80% chance of receiving Outcome B1 (15, 15) if they chose Option B. Under the negative information condition, participants were informed that there was a 20% chance of receiving Outcome B1 (15, 15) if they chose Option B.

Prior to Study 2, a pre-experiment was conducted to validate the effectiveness of manipulating “positive information” and “negative information” on participants. The rationale behind this was that if participants’ initial assessment of the likelihood of obtaining a payoff of (15, 15) was below 20% or above 80%, the positive or negative information provided in Study 2 would have different interpretations. To ensure a more uniform understanding of the positive and negative information provided, and thereby reducing potential confounds that could arise from varying subjective probability estimations, we conducted a preliminary screening of participants.

During the pre-experiment, participants were randomly assigned to either the natural or social risk condition. They were first informed of the basic rules of the trust game and were asked to assess the probability of obtaining a payoff of (15, 15) if they chose option B. Only participants with a subjective probability estimation of 50% were selected to participate in the formal experiment.

According to the results, in the natural risk condition, approximately 63.6% of the participants perceived the subjective risk as 0.5, with a mean degree of adventure 5.92 ± 1.72. 18.2% participants perceived the risk to be higher than 0.5, while the remaining 18.2% believed it to be lower than 0.5. In the social risk condition, 26.6% of the participants perceived the risk as 0.5, with a mean degree of adventure 5.01 ± 1.58. Among the remaining participants, 10.9% believed that the likelihood of the other party selecting (15, 15) was higher than 50%, while 62.5% thought that the likelihood of the other party choosing (15, 15) was lower than 0.5. The final sample included 77 participants in the natural risk condition and 71 participants in the social risk condition.

### 3.2. Formal experiment

#### 3.2.1. Method

##### 3.2.1.1. Participants

The study consisted of a sample of 148 Chinese college students (67 males and 81 females). The mean age was 20.70 (*SD* = 1.23) years, ranging from 18 to 22 years. The exclusion criteria are (1) students major in psychology or economics and (2) had participated in similar decision-making tasks or study. Participants were provided with a small gift as a token of appreciation upon completion of the experiment.

##### 3.2.1.2. Design

The study employed a 2 (risk source: natural vs. social) × 2 (information valence: positive vs. negative) between-subjects design to investigate the effect of descriptive information on risk perception and adventure. The information valence factor here were manipulated through the perceived likelihood of positive information in the risky option in Trust Game, with positive information an 80% chance of obtaining (15, 15) and negative information a 20% chance of obtaining (15, 15).

##### 3.2.1.3. Procedures and materials

Study 2 utilized a one-shot trust game task that was similar to that of Study 1. The participants are clearly informed of the possible outcomes of choosing option A or option B. Before making their decisions, participants were provided with the probability information about the likelihood of obtaining a positive outcome (15, 15) if option B was chosen. Following the probability presentation, participants proceeded to the trust game, where they evaluated risks and made decisions. Half of the participants were required to make their decision before evaluating the risks, while the other half made their decision after completing the risk assessment process.

Natural-Positive Condition. The participants were informed, “There is an 80% possibility that you would draw the result of (15, 15) if you select choice B.” This condition was randomly allocated to 41 participants in the natural risk condition experiment who assessed the outcome probability as 50% during the pre-experiment.

Natural-Negative Condition. The participants were informed, “There is an 20% possibility that you would draw the result of (15, 15) if you select choice B.” This condition was randomly allocated to 36 participants in the natural risk condition experiment who assessed the outcome probability as 50% during the pre-experiment.

Social-Positive Condition. The participants were informed, “According to earlier study, there is an 80% chance that the other party will select (15, 15) if you select choice B.” This condition was randomly allocated to 38 participants in the social risk condition experiment who assessed the outcome probability as 50% during the pre-experiment.

Social-Negative Condition. The participants were informed, “According to earlier study, there is an 20% chance that the other party will select (15, 15) if you select choice B.” This condition was randomly allocated to 33 participants in the social risk condition experiment who assessed the outcome probability as 50% during the pre-experiment.

##### 3.2.1.4. Outcome variables

Similar to Study 1, two outcomes were measured in Study 2: changes in self-reported risk perception and changes in adventure compared to pre-experiment. Adventure was evaluated on a 9-point scale, where 1 indicated a complete preference for option A, 5 indicated neutrality, and 9 indicated a complete preference for option B. Higher scores indicated a greater degree of adventure. Risk perception was evaluated based on participants’ subjective probability estimation of outcome B1 (15, 15) if they chose option B, with values ranging from 0 to 1. A higher value indicated a lower risk perception. Participants were asked to assess their likelihood of obtaining a positive outcome in this specific round to distinguish risk perception from externally provided probabilities.

The change in adventure and risk perception was calculated by comparing the results from the pre-experiment and the formal experiment. The impact of descriptive information in the positive condition was determined by subtracting the degree of adventure in the pre-experiment from the formal experiment, while the change in risk perception was calculated by subtracting 0.5 from the risk perception value in the formal experiment. Similarly, the effect of descriptive information in negative condition was determined by subtracting the adventure degree in pre-experiment from the formal experiment, and the change in risk perception was calculated by subtracting the risk perception in the formal experiment by 0.5.

#### 3.2.2. Results

Table 2 presents the results of the study regarding the influence of descriptive information on adventure and risk perception in the context of social and natural risks.

**Table 2:** Individuals’ adventure and risk perception under four experimental conditions.

	Adventure	Risk perception
natural-positive condition	6.39 ± 0.17	0.77 ± 0.12
natural-negative condition	4.25 ± 1.73	0.32 ± 0.16
social-positive condition	5.74 ± 1.37	0.68 ± 0.15
social-negative condition	4.18 ± 1.21	0.40 ± 0.13

**Figure 3:**
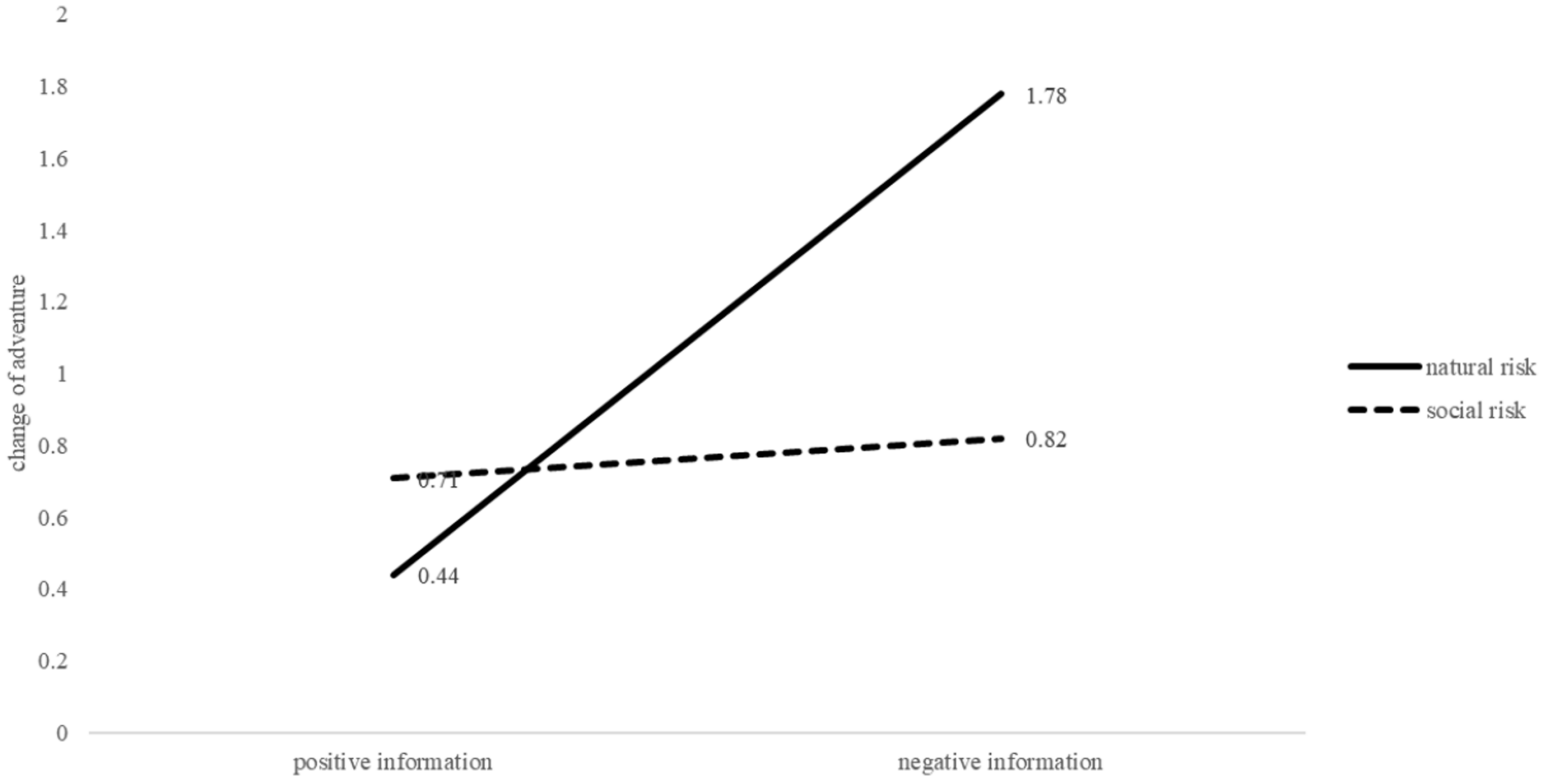
Change of adventure under natural and social condition after receiving positive or negative information.

**Figure 4:**
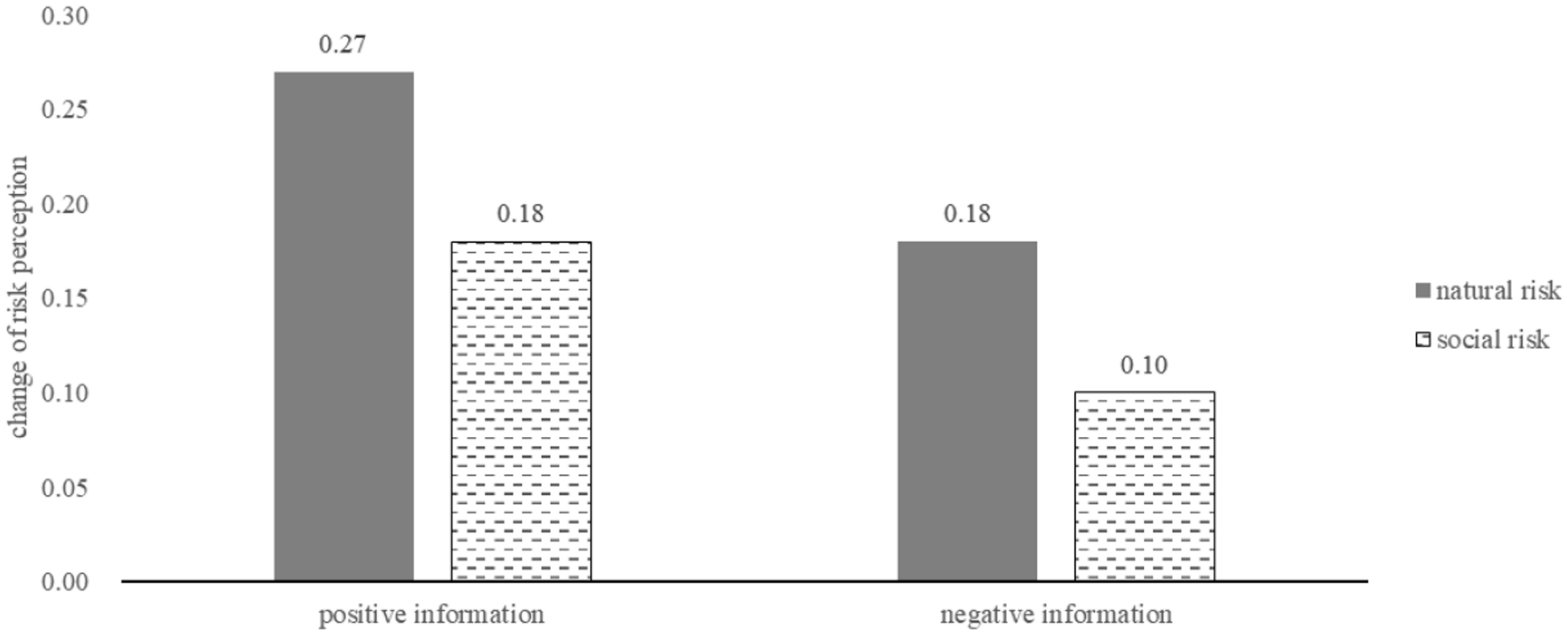
Change of risk perception under natural and social condition after receiving positive or negative information.

The change of adventure and change of risk perception were presented in Figures 3, 4.

In the context of social risks, the effect of information valence on adventure was not found to be significant, *t* (69) = −0.33, *p* > 0.05. This finding supports Hypothesis 3, which suggests that descriptive information has a symmetrical impact on social risks. However, there was a significant asymmetrical effect on risk perception, *t* (69) = 2.29, *p* = 0.024.

In the context of natural risk, the study found that descriptive information had a significant effect on both adventure and risk perception. The impact was asymmetrical, with negative information having a greater effect in decreasing adventure than positive information had in increasing it, *t* (75) = −3.09, *p* = 0.003. Furthermore, there was a significant change in risk perception, *t* (75) = 2.59, *p* = 0.011. These findings contradict Hypothesis 4, which proposed that the impact of descriptive information on natural risk would be symmetrical.

The influence of negative information on adventure differed significantly between natural and social risks, with natural risks showing a greater impact than social risks, which supports Hypothesis 6, *t* (67) = 2.25, *p* = 0.028. This suggests that the asymmetry under descriptive information is greater in natural risk than in social risk. However, the study did not find any significant difference in the impact of positive information on adventure between natural and social risks.

The study employed the intermediary effect analysis procedure proposed by Zhao et al. (2010) and the Bootstrap method proposed by Preacher and Hayes (2004) and Hayes and Scharkow (2013) to examine the role of risk perception change as an intermediary variable in the relationship between information valence and risk-taking behavior. Results showed that, at a 95% confidence interval, the intermediary effect of the change in risk perception was significant (with an interval did not include 0, LLCI = −0.485, ULCI = −0.051), with a size of −0.211. Additionally, after controlling for the change in risk perception, the effect of information valence on the change in risk-taking remained significant, with an interval (LLCI = 0.403, ULCI = 1.517) that did not include 0. These results suggest that the change in risk perception plays a partial intermediary role in the impact of information valence on the change in risk-taking behavior. The results of the study provide evidence to support Hypothesis 7 in the context of descriptive information.

## 4. Discussion

Firstly, we conducted study on the trust asymmetry in natural and social risk contexts, it theoretically broadened the literature of trust asymmetry and innovated traditional research methods. Prior research primarily focused on investigating trust asymmetry in complex risk scenarios and treated risks as a singular construct (Slovic, 1993; O'Brien et al., 2021). However, this approach may not be sufficient in fully understanding the complexity and variability of risk decision in different contexts. From a practical standpoint, such research conclusions may have generalization limitations because they limit understanding of the complexity of risk and do not adequately explain the inconsistent results of studies of trust asymmetry in different contexts. In the current risk society, individual’s concerns about technological risks include the reliability of science and technology itself, as well as risks arising from people’ abuse and misuse. In our research, we specified the types of risk and compared natural risk to social risk, enriching the environment conditions for trust asymmetry research. The findings did unpack the difference between natural and social risk sources on trust asymmetrical, highlighting the study’s significant theoretical and practical value.

Second, trust issues often arise due to negative experiences, and efforts to restore trust may involve the disclosure of comprehensive safety and quality data. However, previous research has primarily focused on the impact of experiential information on trust. Our study supplements the consideration and emphasis on descriptive and experiential information, theoretically respond to the call for diverse information considerations, and greatly supplements the lack of application and promotion in this field in practice.

Third, the present study differs from previous research on trust asymmetry by employing a measurement of both trust behavior and risk perception as the dependent variable, rather than relying solely on self-reported measures of trust. This methodological approach contributes to the methodological diversity of the field and provides a more objective measure of trust behavior.

### 4.1. Descriptive versus experiential information on trust vulnerability

Cvetkovich and Winter (2004) conducted a study on the impact of information specificity on trust asymmetry and found that the asymmetry is less prominent for low specificity information, such as risk management policies, compared to high specificity information, such as specific events. However, it is important to note that the low specificity information used in their study was limited to risk managers’ policies, which pertain to intentions and processes, whereas specific events are actual outcomes that may impact trust through different dimensions. Additionally, low specificity information with high diagnostic value may have a broader scope than policies. Therefore, the generalizability of their results in terms of information specificity needs to be carefully considered. In this study, we introduce the concepts of descriptive and experiential information to further investigate the relationship between information type and trust asymmetry.

The results of the study indicate that the asymmetry of trust varies with the types of information. Trust is found to be more vulnerable under experiential information, where negative information has a greater impact on reducing trust compared to positive information increasing it. In contrast, trust is more resilient under descriptive information, where the impact of positive and negative information is similar.

We contend that the varying effects of trust asymmetries in descriptive versus experiential decision-making can be attributed to differences in processing and representation styles for each type of information. Experiential decision-making is characterized by implicit and emotional processing mechanisms, while descriptive decision-making relies on explicit and cognitive processing mechanisms (Chang et al., 2010). In situations where trust has been breached, the negative emotional impact of betrayal can result in mental losses that outweigh the impact of material losses. As the saying goes, “fool me once, shame on you; fool me twice, shame on me,” negative experiences have a more significant impact on social risk perception than positive experiences, leading to trust asymmetries. This psychological effect can significantly increase the costs associated with restoring trust, as even small negative encounters can significantly diminish trust levels. To rebuild trust, it is necessary to introduce positive experiences with enough intensity to offset the negative experiences and decrease the sense of risk. On the contrary, descriptive information provides a comprehensive and summary description of the probability of outcomes, with minimal influence from emotional biases during the processing stage. Consequently, positive and negative descriptive information offers similar diagnostic value and impact on social risk perception for individuals, leading to more symmetrical decision-making.

Under descriptive and experiential contexts, individuals both exhibit different patterns under natural risks compared to social risks. Empirical evidence suggests that positive and negative experiential information make symmetrical impacts on natural risk perception. This may be attributed to the notion that a singular feedback is insufficient to significantly impact individuals’ natural risk perception, thus resulting in similar effects for positive and negative information. However, regarding descriptive information, it has been observed that negative information has a more substantial impact on adventure than positive information, which is inconsistent with our hypothesis. We suggest the need of considering the absolute value of adventure, rather than just the relative values that can change after receiving a message. While positive descriptive information has a limited effect on enhancing natural risk-taking, the highest absolute value of risk-taking behavior was observed under the “natural risk-positive information” condition among the four experimental treatments in Study 2. This may suggest that the effect of increased descriptive information on adventure follows a diminishing slope, where the impact of positive information diminishes as the level of adventure reaches a certain threshold. Further investigation is necessary to confirm this hypothesis.

### 4.2. Comparison of asymmetry between natural and social risks

The study also investigates trust as a kind of social risks. Specifically, the study aims to determine whether negative information has a greater impact on social risks compared to natural risks under same conditions. The results of the study suggest that negative descriptive information has a more significant impact on natural risks, whereas negative experiential information has a greater impact on social risks.

The influence of negative descriptive information is greater on decision making related to natural risks as compared to social risks. In other words, social risky decision making seems to be more resistant to negative descriptive information. This phenomenon may be attributed to the lower diagnostic value of descriptive information for social risks in comparison to natural risks. People tend to establish a stable sense of trustworthiness based on their prior experiences, which serves as a crucial reference point for their decision-making. Thus, the perception of social risk is influenced by both past experiences and external descriptive information (Bellucci and Park, 2020). Conversely, individuals tend to view natural risk as a random event, with the magnitude of risk perception being anchored by descriptive information.

Social risk is more responsive to negative information compared to natural risk when individuals are making decisions based on experience. This is due to the psychological losses that result from experiences of betrayal in social risk scenarios. Even a small amount of negative information can significantly impact risk perception in social risk context. In contrast, a few experiences are typically not sufficient to significantly alter individuals’ perceptions of natural risks, which are often perceived as independent and characterized by fixed probabilities. Studies on recency effects, including positive effects like the “hot hand” phenomenon and negative effects like the “gambler’s fallacy,” suggest that sustained and consistent results are necessary to elicit cognitive biases (Tversky and Kahneman, 1981; Clotfelter and Cook, 1993). Nonetheless, it remains unclear whether the impact of experiential information on asymmetry changes with an increase in feedback information to a certain threshold, as demonstrated in many studies exploring descriptive-experiential disparities (Harman et al., 2019). Further research is necessary to investigate this phenomenon.

### 4.3. Potential neural mechanisms

While this study did not directly examine neural mechanisms, we draw upon previous research investigating the physiological underpinnings of natural and social risks to explore potential neural mechanisms associated with trust asymmetry in descriptive and experiential decision-making.

Currently, there is a dearth of neurophysiological studies specifically investigating the neural underpinnings of trust asymmetry. However, research in the neural mechanisms of betrayal aversion offers valuable insights in this regard. Research suggests that increased activation in the insula is a crucial neural factor associated with betrayal aversion, and the negative emotions related to betrayal or unfair treatment, rather than risk aversion, conveyed by the insula, are the source of behavioral disparities between natural and social risk decisions (Aimone et al., 2014).

While the insula has been implicated in processing various forms of risk and uncertainty, including natural and social risks (Elliott et al., 2000; Paulus et al., 2003; King-Casas et al., 2008), its activation is particularly pronounced when making decisions entailing potential betrayal compared to natural risk, leading to a reduced inclination for beneficial social interactions (Aimone et al., 2014). Other studies also support the role of the insula in (dis) trust. The insula shows increased activation as the level of distrust increases (Winston et al., 2002; Kragel et al., 2015), encounters with betrayal elicit greater activation in the left anterior insula (van den Bos et al., 2011), individuals with insula damage tend to exhibit more trusting behavior (Belfi et al., 2015), and the right anterior insula plays a critical role in distinguishing “us” from “them” (Lau et al., 2020). These findings collectively suggest that the insula may serve as a crucial neurophysiological foundation for understanding trust asymmetry phenomena.

The *ventromedial prefrontal cortex* (vmPFC) may also play a crucial role in trust asymmetry. The vmPFC exhibits a close association with trust levels, as evidenced by a positive correlation between individuals’ trust tendencies and gray matter volume within the vmPFC region (Haas et al., 2015). Additionally, individuals with vmPFC damage demonstrate an inability to adjust their behavior based on interactive feedback (Moretto et al., 2013), suggesting a potential absence of trust asymmetry. Thus, it can be inferred that vmPFC activation may constitute one of the neural mechanisms underlying trust asymmetry. Moreover, the vmPFC may be related to the descriptive-experiential gap in trust asymmetry according to the current findings. It is important to note that the influence of the vmPFC may be confined to experiential decision-making rather than affecting descriptive decision-making. FitzGerald and colleagues found significantly lower vmPFC activity in descriptive decision-making compared to experiential decision-making (FitzGerald et al., 2010). Similarly, in a study employing the Iowa gambling task (IGT), Fellows and Farah observed that individuals with damage to the vmPFC demonstrated deficits in experiential learning, as opposed to the conventional phenomenon of loss aversion (Fellows and Farah, 2003). Higher vmPFC activation in experiential decision-making and decreased activation in descriptive decision-making, these neuroscience-related findings align with the descriptive-experiential gap in trust asymmetry within this study. Moreover, researchers have found that the *ventrolateral prefrontal cortex* (vlPFC) maintains choices linked to reliable prior beliefs when prior information is present in descriptive decision-making scenarios (Fouragnan et al., 2013), indirectly supporting the resilience of trust in descriptive decision-making.

The insula and vmPFC are important neural mechanisms that could be key areas of focus in future research on trust asymmetry. Additionally, several neurochemical factors associated with trust and decision-making, such as oxytocin (Kosfeld et al., 2005), testosterone (Boksem et al., 2013), the noradrenergic system (Rogers et al., 2004), and blood glucose (Wang, 2018), may also play significant roles in trust asymmetry and warrant further investigation. In the current study, trust asymmetry was not observed within the context of descriptive decision-making. It is hypothesized that this absence of asymmetry may be attributed to individuals relying on their own past experiences in similar situations when assessing trustworthiness. To support this hypothesis, future investigations could examine the activation patterns of brain regions associated with contextual memory processing, such as the hippocampus and parahippocampal gyrus, in relation to descriptive decision-making involving both natural and social risks.

## 5. Limitations

One important limitation of this study is the potential lack of generalizability of the experimental tasks and scenarios employed. It is crucial to admit that trust vulnerability is a context-specific phenomenon. However, the study only examined decision-making asymmetry in gain scenarios, whereas prospect theory suggests that individuals exhibit distinct decision-making preferences when dealing with gains versus losses.

Moreover, the study assessed the impact of experiential information on trust asymmetry using a two-round game task that provided the outcome of a single decision, resulting in a less immersive information environment than continuous stochastic decision-making. In reality, individuals often rely on the accumulation of experiences from multiple exploratory decisions. Researchers have uncovered variations in individuals’ decision-making behavior between long-term and short-term interactions. Notably, long-term decision-making involving natural risks exhibits some subtle dynamic interactions (Lebiere et al., 2007), and risk aversion biases can be mitigated through ample sampling. Additionally, as the level of information increases, there is a corresponding rise in individual cooperation and mutual cooperation (Martin et al., 2014), suggesting that long-term trust interactions may surmount short-term optimization challenges in social interactions, ultimately diminishing trust asymmetry. Consequently, future investigations should expand the available dataset within multi-round game scenarios and develop mathematical or computational models to provide a comprehensive understanding of the asymmetry issues surrounding natural and social risks across diverse interaction contexts.

A related limitation of this study is its exclusive reliance on a trust game with monetary incentives as the primary decision-making focus. The effects of smaller monetary incentives on individuals’ arousal levels and subsequent decision-making behaviors may diverge from those related to safety and health concerns. Therefore, the generalizability of the findings to other contexts and domains beyond monetary incentives, such as safety and health concerns, requires further investigation to ensure ecological validity.

Furthermore, this study is traditional behavioral research that employs relatively small-scale interpretable models containing only a few explanatory variables. However, it is important to note that this approach may be limited by the “flatland fallacy,” which restricts scientists’ capacity to build and communicate useful models of human psychology (Jolly and Chang, 2019). In future research, it is essential to investigate the damage and restoration processes of trust from a high-dimensional perspective, such as Intuitive Mental Model (Suomala and Kauttonen, 2022).

An additional limitation of the study was that it posited a crucial role for emotions in the implicit information processing elicited by experiential information. However, the study did not thoroughly examine the impact of different emotional states on risk perception and decision-making asymmetry. Moreover, the role of “value,” another crucial variable that can influence risk decision-making, remained unexplored in this study. Further research is necessary to investigate these issues in greater depth.

The study was also limited by its scope of application. The research focused solely on the impact of descriptive and experiential information on trust vulnerability. In real-life situations, information scenarios often involve a combination of different types of information. Further investigation is necessary to determine whether and how positive descriptive information repairs trust after negative experiential information damages it.

## 6. Conclusion

This study investigated trust asymmetry from two distinct perspectives: the asymmetry of trust damage and enhancement in response to positive and negative information, and the asymmetry of the negative impact of negative information on natural and social risks. When exposed to experiential information, trust was found to be more susceptible to negative information compared to positive information, resulting in a higher level of vulnerability. Conversely, when exposed to descriptive information, trust was relatively symmetry and showing less susceptibility to negative information compared to natural risks, leading to a higher degree of resilience. The study suggests that trust asymmetry and repair must be tailored to the specific situation. Additional research is required to further investigate these findings.

## Data availability statement

The raw data supporting the conclusions of this article will be made available by the authors, without undue reservation.

## Ethics statement

This study was carried out in accordance with the Declaration of Helsinki. Electronic informed consent was obtained from all participants before the survey. The research protocol was approved by the Research Ethics Review Committee of Xiamen University (No. XDYX202305K27).

## Author contributions

JZ designed the study, performed the analysis and interpreted the data, gathered the data, and drafted the manuscript. JZ, YY, and SJ revised the manuscript. All authors contributed to the article and approved the submitted version.

## Funding

This study was supported by Social Science Fund of Fujian Province (grant no. FJ2022B144) and the Fundamental Research Funds for the Central Universities (grant no. 2072021143).

## Conflict of interest

The authors declare that the research was conducted in the absence of any commercial or financial relationships that could be construed as a potential conflict of interest.

## Publisher’s note

All claims expressed in this article are solely those of the authors and do not necessarily represent those of their affiliated organizations, or those of the publisher, the editors and the reviewers. Any product that may be evaluated in this article, or claim that may be made by its manufacturer, is not guaranteed or endorsed by the publisher.

## References

[ref1] Aimone J. A. Houser D. Weber B. (2014). Neural signatures of betrayal aversion: an fMRI study of trust. P Roy. Soc. B-Biol. Sci. 281:20132127. doi: 10.1098/rspb.2013.2127 PMC3973250 24648217

[ref2] Beck U. (1992). Modern Society as a Risk society: Inquiries into Contemporary Societies. Nanjing: Yilin Publishing House.

[ref3] Belfi A. M. Koscik T. R. Tranel D. (2015). Damage to the insula is associated with abnormal interpersonal trust. Neuropsychologia 71, 165–172. doi: 10.1016/j.neuropsychologia.2015.04.003, PMID: 25846668 PMC4417431

[ref4] Bellucci G. Park S. Q. (2020). Honesty biases trustworthiness impressions. J. Exp. Psychol. Gen. 149, 1567–1586. doi: 10.1037/xge0000730 31916837

[ref5] Bohnet I. Greig F. Herrmann B. Zeckhauser R. (2008). Betrayal aversion: evidence from Brazil, China, Oman, Switzerland, Turkey, and the United States. Am. Econ. Rev. 98, 294–310. doi: 10.1257/aer.98.1.294

[ref6] Boksem M. A. S. Mehta P. H. Van den Bergh B. van Son V. Trautmann S. T. Roelofs K.. (2013). Testosterone inhibits trust but promotes reciprocity. Psychol. Sci. 24, 2306–2314. doi: 10.1177/0956797613495063, PMID: 24071565

[ref7] Chang L. J. Doll B. B. Wout M V. T. Frank M. J. Sanfey A. G. (2010). Seeing is believing: trustworthiness as a dynamic belief. Cognitive Psychol. 61, 87–105. doi: 10.1016/j.cogpsych.2010.03.001, PMID: 20553763

[ref8] Clotfelter C. T. Cook P. J. (1993). The “gambler's fallacy” in lottery play. Manag. Sci. 39, 1521–1525. doi: 10.1287/mnsc.39.12.1521

[ref9] Cvetkovich G. Siegrist M. Murray R. Tragesser S. (2002). New information and social trust: asymmetry and perseverance of attributions about hazard managers. Risk Anal. 22, 359–367. doi: 10.1111/0272-4332.00030, PMID: 12022682

[ref10] Cvetkovich G. T. Winter P. L. Seeing Eye-to-Eye on Natural Resource Management: Trust, Value Similarity, and Action Consistency/Justification: San Francisco State University; San Francisco, CA (2004).

[ref11] De Jong B. A. Dirks K. T. (2012). Beyond shared perceptions of trust and monitoring in teams: implications of asymmetry and Dissensus. J. Appl. Psychol. 97, 391–406. doi: 10.1037/a0026483, PMID: 22181679

[ref12] Elliott R. Friston K. J. Dolan R. J. (2000). Dissociable neural responses in human reward systems. J. Neurosci. 20, 6159–6165. doi: 10.1523/JNEUROSCI.20-16-06159.2000, PMID: 10934265 PMC6772605

[ref13] Falk R. Konold C. (1997). Making sense of randomness: implicit encoding as a basis for judgment. Psychol. Rev. 104, 301–318. doi: 10.1037/0033-295X.104.2.301

[ref14] Fellows L. K. Farah M. J. (2003). Ventromedial frontal cortex mediates affective shifting in humans: evidence from a reversal learning paradigm. Brain 126, 1830–1837. doi: 10.1093/brain/awg180, PMID: 12821528

[ref15] Fetchenhauer D. Lang A. S. Ehlebracht D. Schlosser T. Dunning D. (2020). Does betrayal aversion really guide trust decisions towards strangers? J. Behav. Decis. Making. 33, 556–566. doi: 10.1002/bdm.2166

[ref16] FitzGerald T. H. B. Seymour B. Bach D. R. Dolan R. J. (2010). Differentiable neural substrates for learned and described value and risk. Curr. Biol. 20, 1823–1829. doi: 10.1016/j.cub.2010.08.048, PMID: 20888231 PMC2977067

[ref17] Fouragnan E. Chierchia G. Greiner S. Neveu R. Avesani P. Coricelli G. (2013). Reputational priors magnify striatal responses to violations of trust. J. Neurosci. 33, 3602–3611. doi: 10.1523/JNEUROSCI.3086-12.2013, PMID: 23426687 PMC6619519

[ref18] Garcia B. Cerrotti F. Palminteri S. (2021). The description-experience gap: a challenge for the neuroeconomics of decision-making under uncertainty. Philos T R Soc B. 376:20190665. doi: 10.1098/rstb.2019.0665, PMID: 33423626 PMC7815421

[ref19] Haas B. W. Ishak A. Anderson I. W. Filkowski M. M. (2015). The tendency to trust is reflected in human brain structure. NeuroImage 107, 175–181. doi: 10.1016/j.neuroimage.2014.11.060 25485710

[ref20] Harman J. L. Zhang D. Greening S. G. (2019). Basic processes in dynamic decision making: how experimental findings about risk, uncertainty, and emotion can contribute to police decision making. Front. Psychol. 10:10. doi: 10.3389/fpsyg.2019.02140 31620062 PMC6763579

[ref21] Hau R. Pleskac T. J. Hertwig R. (2010). Decisions from experience and statistical probabilities: why they trigger different choices than a priori probabilities. J. Behav. Decis. Making. 23, 48–68. doi: 10.1002/bdm.665

[ref9001] Hayes A. F. Scharkow M. (2013). The relative trustworthiness of inferential tests of the indirect effect in statistical mediation analysis: does method really matter?. Psychological science 24, 1918–1927. 23955356 10.1177/0956797613480187

[ref22] Hertwig R. Barron G. Weber E. U. Erev I. (2004). Decisions from experience and the effect of rare events in risky choice. Psychol. Sci. 15, 534–539. doi: 10.1111/j.0956-7976.2004.00715.x, PMID: 15270998

[ref23] Humphrey S. J. Mondorf S. (2021). Testing the causes of betrayal aversion. Econ. Lett. 198:109663. doi: 10.1016/j.econlet.2020.109663

[ref24] Incekara-Hafalir E. Kim E. Stecher J. D. (2021). Is the Allais paradox due to appeal of certainty or aversion to zero? Exp. Econ. 24, 751–771. doi: 10.1007/s10683-020-09678-4

[ref25] Jolly E. Chang L. J. (2019). The flatland fallacy: moving beyond low-dimensional thinking. Top. Cogn. Sci. 11, 433–454. doi: 10.1111/tops.12404, PMID: 30576066 PMC6519046

[ref26] Kahneman D. Tversky K. A. (1979). Prospect theory: an analysis of decision under risk. Econometrica 47, 263–291. doi: 10.2307/1914185

[ref27] King-Casas B. Sharp C. Lomax-Bream L. Lohrenz T. Fonagy P. Montague P. R. (2008). The rupture and repair of cooperation in borderline personality disorder. Science 321, 806–810. doi: 10.1126/science.1156902, PMID: 18687957 PMC4105006

[ref28] Klinke A. Renn O. (2021). The coming of age of risk governance. Risk Anal. 41, 544–557. doi: 10.1111/risa.13383 31379003

[ref29] Kosfeld M. Heinrichs M. Zak P. J. Fischbacher U. Fehr E. (2005). Oxytocin increases trust in humans. Nature 435, 673–676. doi: 10.1038/nature03701 15931222

[ref30] Kragel P. A. Zucker N. L. Covington V. E. LaBar K. S. (2015). Developmental trajectories of cortical-subcortical interactions underlying the evaluation of trust in adolescence. Soc. Cogn. Affect. Neur. 10, 240–247. doi: 10.1093/scan/nsu050, PMID: 24682131 PMC4321625

[ref31] Lane J. N. (2023). The subjective expected utility approach and a framework for defining project risk in terms of novelty and feasibility - a response to Franzoni and Stephan (2023), uncertainty and risk-taking in science. Res. Policy 52:104707. doi: 10.1016/j.respol.2022.104707

[ref32] Lau T. Gershman S. J. Cikara M. (2020). Social structure learning in human anterior insula. elife 9:9. doi: 10.7554/eLife.53162 PMC7136019 32067635

[ref33] Lebiere C. Gonzalez C. Martin M. Instance-based decision-making model of repeated binary choice. In proceedings of the 8th international conference on cognitive Modeling. (2007).

[ref34] Martin J. M. Gonzalez C. Juvina I. Lebiere C. (2014). A description-experience gap in social interactions: information about interdependence and its effects on cooperation. J. Behav. Decis. Making. 27, 349–362. doi: 10.1002/bdm.1810

[ref35] Moretto G. Sellitto M. di Pellegrino G. (2013). Investment and repayment in a trust game after ventromedial prefrontal damage. Front. Hum. Neurosci. 7:593. doi: 10.3389/fnhum.2013.00593, PMID: 24093013 PMC3782646

[ref36] Nobandegani A. S. Shultz T. Dubé L. A unified, resource-rational account of the Allais and Ellsberg paradoxes. In proceedings of the annual meeting of the cognitive science society. (2021) 43

[ref37] O'Brien T. C. Palmer R. Albarracin D. (2021). Misplaced trust: when trust in science fosters belief in pseudoscience and the benefits of critical evaluation. J. Exp. Soc. Psychol. 96:104184. doi: 10.1016/j.jesp.2021.104184 PMC13004600 41867879

[ref38] Paulus M. P. Rogalsky C. Simmons A. Feinstein J. S. Stein M. B. (2003). Increased activation in the right insula during risk-taking decision making is related to harm avoidance and neuroticism. NeuroImage 19, 1439–1448. doi: 10.1016/S1053-8119(03)00251-9, PMID: 12948701

[ref39] Payzan-LeNestour E. Doran J. Craving for Money. Elsevier BV. Amsterdam (2022).

[ref40] Poortinga W. Pidgeon N. F. (2004). Trust, the asymmetry principle, and the role of prior beliefs. Risk Anal. 24, 1475–1486. doi: 10.1111/j.0272-4332.2004.00543.x, PMID: 15660605

[ref9003] Preacher K. J. Hayes A. F. (2004). SPSS and SAS procedures for estimating indirect effects in simple mediation models. Behavior research methods, instruments, & computers, 36, 717–731. 10.3758/bf03206553 15641418

[ref41] Ran B. Qi H. T. (2019). The entangled twins: power and Trust in Collaborative Governance. Admin Soc. 51, 607–636. doi: 10.1177/0095399718801000

[ref42] Rogers R. D. Ramnani N. Mackay C. Wilson J. L. Jezzard P. Carter C. S.. (2004). Distinct portions of anterior cingulate cortex and medial prefrontal cortex are activated by reward processing in separable phases of decision-making cognition. Biol. Psychiatry 55, 594–602. doi: 10.1016/j.biopsych.2003.11.012, PMID: 15013828

[ref43] Schmeidler D. (1989). Subjective-probability and expected utility without additivity. Econometrica 57, 571–587. doi: 10.2307/1911053

[ref44] Siegrist M. Cvetkovich G. (2001). Better negative than positive? Evidence of a bias for negative information about possible health dangers. Risk Anal. 21, 199–206. doi: 10.1111/0272-4332.211102, PMID: 11332549

[ref45] Slovic P. (1993). Perceived risk, trust, and democracy. Risk Anal. 13, 675–682. doi: 10.1111/j.1539-6924.1993.tb01329.x

[ref46] Suomala J. Kauttonen J. (2022). Human's intuitive mental models as a source of realistic artificial intelligence and engineering. Front. Psychol. 13:13. doi: 10.3389/fpsyg.2022.873289 PMC9189375 35707640

[ref9002] Taylor S. E. (1991). Asymmetrical effects of positive and negative events: The mobilizationminimization hypothesis. Psychological Bulletin 110:6. 10.1037/0033-2909.110.1.67 1891519

[ref47] Thaler R. H. Johnson E. J. (1990). Gambling with the house money and trying to break even - the effects of prior outcomes on risky choice. Manag. Sci. 36, 643–660. doi: 10.1287/mnsc.36.6.643

[ref48] Tversky A. Kahneman D. (1981). The framing of decisions and the psychology of choice. Science 211, 453–458. doi: 10.1126/science.7455683 7455683

[ref49] van den Bos W. van Dijk E. Westenberg M. Rombouts S. A. R. B. Crone E. A. (2011). Changing brains, changing perspectives: the neurocognitive development of reciprocity. Psychol. Sci. 22, 60–70. doi: 10.1177/0956797610391102, PMID: 21164174

[ref51] Wang M. Y. Zhang Q. Y. Zhou K. Z. (2020). The origins of trust asymmetry in international relationships: an institutional view. J. Int. Mark. 28, 81–101. doi: 10.1177/1069031X19898492

[ref50] Wang X. T. (2018). Resource Signaling via blood glucose in embodied decision making. Front. Psychol. 9:9. doi: 10.3389/fpsyg.2018.01965 30374322 PMC6196271

[ref52] Weber E. U. (2006). Experience-based and description-based perceptions of long-term risk: why global warming does not scare us. Clim. Chang. 77, 103–120. doi: 10.1007/s10584-006-9060-3

[ref53] Winston J. S. Strange B. A. O'Doherty J. Dolan R. J. (2002). Automatic and intentional brain responses during evaluation of trustworthiness of faces. Nat. Neurosci. 5, 277–283. doi: 10.1038/nn816, PMID: 11850635

[ref54] Wulff D. U. Mergenthaler-Canseco M. Hertwig R. (2018). A Meta-analytic review of two modes of learning and the description-experience gap. Psychol. Bull. 144, 140–176. doi: 10.1037/bul0000115, PMID: 29239630

[ref55] Zhao X. S. Lynch J. G. Chen Q. M. (2010). Reconsidering baron and Kenny: myths and truths about mediation analysis. J. Consum. Res. 37, 197–206. doi: 10.1086/651257

[ref56] Zucker L. G. (1986). Production of trust: institutional sources of economic structure, 1840-1920. Res. Organ. Behav. 8, 53–111.

